# Climate Change and Agricultural Agents: Planning for Future Interactions

**DOI:** 10.1289/ehp.117-a163b

**Published:** 2009-04

**Authors:** Tanya Tillett

Global climate change is expected to cause increasingly extreme oscillations in atmospheric temperatures as well as increased frequencies and intensities of storms and natural disasters. Other effects may be felt in more indirect ways, such as through altered exposures to chemicals and pathogens whose use and spread, respectively, may shift in response to climate change. Researchers now present various scenarios for potential climate change–related shifts in human exposure to agricultural chemicals and pathogens in the United Kingdom **[*EHP*** **117:508–514; Boxall et al.]**.

A number of potentially hazardous agents are associated with agricultural activities, among them pesticides, fertilizers, pharmaceuticals, plant toxins, and pathogenic bacteria and fungi. Evidence suggests these and other agents travel well beyond farming operations through various channels, resulting in potential human exposure. According to the authors, the main routes of human exposure typically are consumption of food and drinking water, with vector, aerial, and direct contact pathways of less importance for the U.K. population.

Changes in chemical use to accommodate altered crop growing cycles, increased production of naturally occurring mycotoxins, and changes in temperature and precipitation all may contribute to more widespread human exposure to agricultural agents. For instance, more or new pesticides and biocides may be required to offset altered pest activity. This, along with localized increases of dissolved phosphorus and nitrate in drain flow and floodwaters, could lead to more overall exposure via waterways.

Dust released into the atmosphere during tilling and harvesting is a key transport pathway for particulate and particle-bound contaminants, and soil dust has already been linked to a range of human health impacts. The authors predict that hotter, drier summers will lead to increased transfer of surface dust into the environment, and air- and waterborne exposures associated with dust release are likely to be of major public health significance in the future.

Although the authors anticipate a rise in human health hazards associated with climate change–driven exposures to agricultural agents, they believe this rise can likely be managed in large part through research and policy changes such as the development of targeted surveillance schemes for monitoring farm-related pathogens and chemicals, and their health effects, as well as the creation of experimental data sets and models for airborne dust transport and other exposure pathways.

## Figures and Tables

**Figure:**
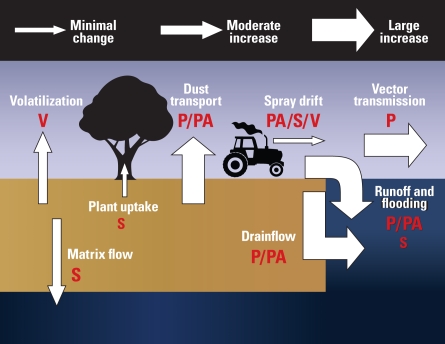
**P = particulate (e.g., microbes, nanoparticles); PA = particle-associated (e.g., hydrophobic organics, ammonium, heavy metals); S = soluble contaminant (e.g., nitrates, hydrophilic pesticides); V = volatile contaminant (e.g., methane). The size of the letter reflects the importance of the pathway to the agent in question.** Source: Boxall ABA et al. *EHP* 117:508–514 (2009).

